# Hepatic Necrosis and Degenerative Myopathy Associated with Cassava Feeding in Pigs

**DOI:** 10.1155/2014/584945

**Published:** 2014-08-12

**Authors:** Gloria Daniel-Igwe

**Affiliations:** Department of Veterinary Pathology, College of Veterinary Medicine, Michael Okpara University of Agriculture, Umudike PMB 7267, Abia State, Nigeria

## Abstract

Forty-three deaths were recorded among pigs fed boiled cassava meal at a private piggery over a period of two years. There were signs of sudden death in some cases with blood exuding from the external nares, vomiting, muscular weakness and pain or reluctance to move, emaciation, and stunted growth. The necropsy lesions included skeletal and cardiac muscle degeneration and necrosis, icterus, hepatic necrosis, and oedema of the dependent parts. The deaths and clinical signs are thought to be due to a non cyanide toxic principle in cassava, possibly the coumarins (scopoletin), which is found in high levels in cassava diet even after heat treatment. Therefore, the use of proper processing technology to obtain cassava products of high quality is recommended.

## 1. Introduction

In Nigeria, commercially reared pigs under the intensive husbandry system are kept in concrete floors and fed balanced commercially produced feeds. The use of cassava (*Manihot utilisima*) in form of the peels and pulp in the feeding of livestock has attracted renewed interest especially with the rising cost of commercially produced feeds [1]. Pig rations are formulated with high energy content to provide for greater growth potential in genetically improved pigs [2]. The private pig farmer fed his pigs only with peeled and boiled cassava pulp as a source of energy and to reduce cost. This paper describes the clinical signs, postmortem lesions, and histopathology of the pigs fed the cassava diet.

## 2. Case Report

Forty-three deaths were recorded among pigs fed boiled cassava meal at a private piggery over a period of two years. A total of thirty-eight deaths occurred among the piglets less than 9 months of age, while only five deaths were reported in the adult pigs over nine months of age. The pigs were of the Landrace × Large White local crossbreeds. On examination, the clinical signs shown by the pigs varied. In acute cases the pigs were found dead without premonitory signs. These pigs were mostly 2–4 months old and in good nutritional body conditions. Blood exuded from the nose and did not clot readily or not at all. In the pigs over one year of age there was a chronic prolonged course of the disease, characterized by lack of interest in their food, progressive emaciation, and stunting; jaundice and recumbency with decubital ulcers were observed in some cases. The abdomen was distended with ascites. The pigs which exhibited clinical symptoms were treated accordingly. However, they failed to respond to treatments and ultimately died. Necropsy lesions in the acute cases showed the liver swollen, pale with focal colour variations. The capsule was coated with fibrin. The lymph nodes were red on cut surface while the spleen was enlarged. The cardiac part of the stomach was ulcerated and covered with diphtheritic membrane. In the chronic cases there were hydrothorax, hydropericardium, oedema of the lungs, and subcutaneous tissues. There was paleness of the skeletal muscles especially of the large muscle masses. The liver was shrunken and fibrotic.

**Figure 1:**
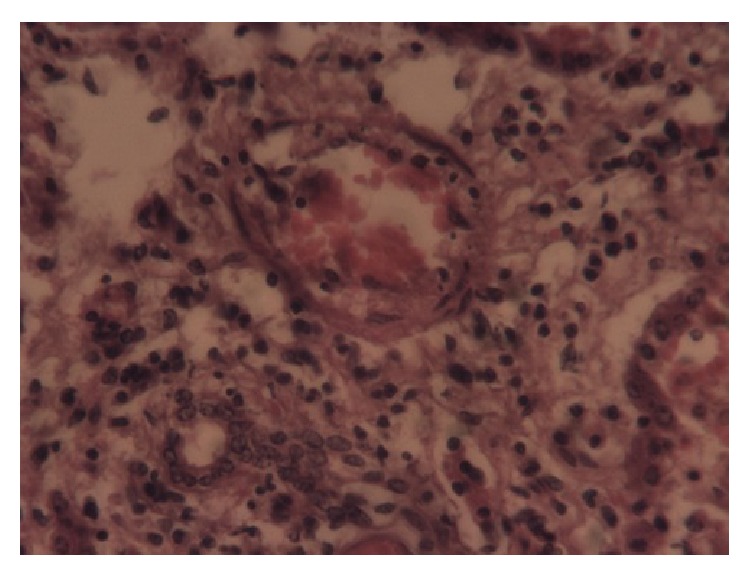
Liver: necrosis and mononuclear cellular infiltration of the liver (He 40x).

**Figure 2:**
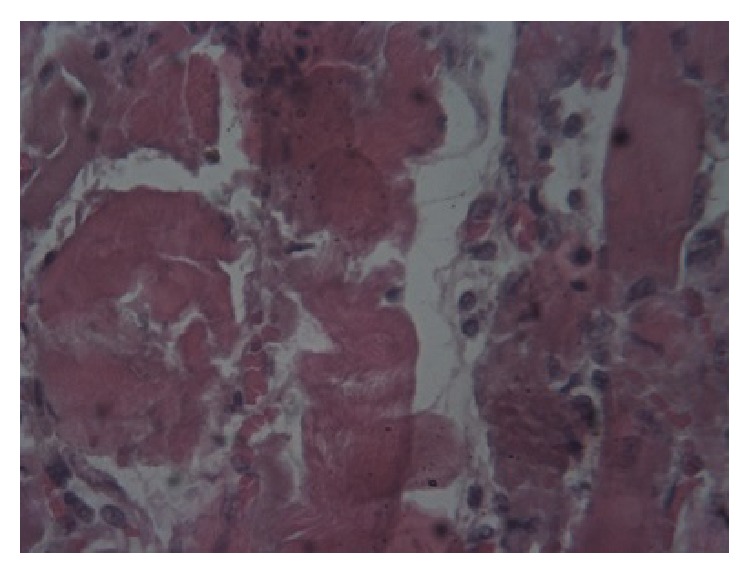
Skeletal muscle: Zenker's degeneration, necrosis of fibres, oedema, and mononuclear cellular infiltration of the skeletal muscle (He 40x).

**Figure 3:**
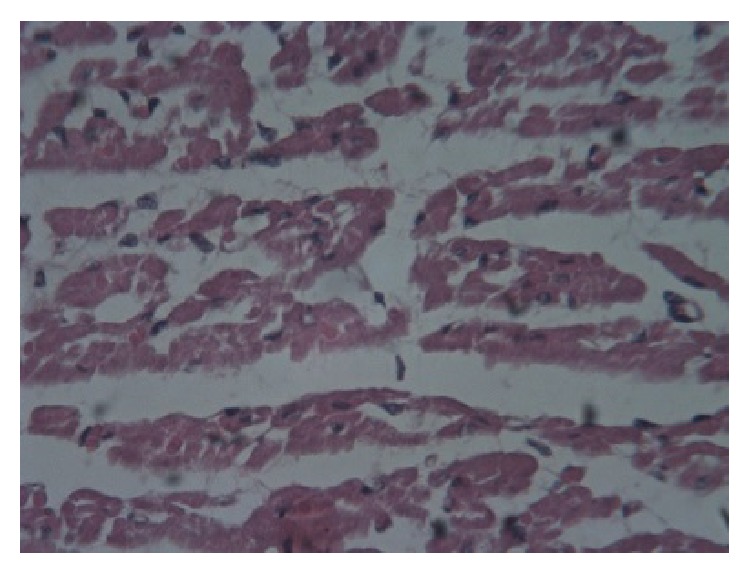
Cardiac muscle: Zenker's degeneration, necrosis of fibres, oedema, and mononuclear cellular infiltration of the cardiac muscle (He 40x).

Postmortem tissues from the liver, heart, and skeletal muscles were fixed in 10% neutral buffered formal saline, embedded in paraffin wax, and thereafter sectioned at 5 *μ*m. The slides were stained using haematoxylin and eosin (H & E) technique. The histopathological examination of the tissues revealed that, in the acute cases, the liver lobules showed spot-like degeneration and coagulative necrosis. In the chronic cases many liver lobules showed postnecrotic collapse and fibrosis, bile duct hyperplasia and scattered partially destroyed lobules, and mononuclear cellular infiltration (Figure 1). The skeletal and cardiac muscles showed Zenker's degeneration and necrosis of the fibres and an increase in mononuclear cells and oedema between the fibres (Figures 2 and 3).

Smears and swabs of tissue fluids from the liver, kidney, and lymph nodes were subjected to microbial isolation tests following standard microbiological procedures [3]. Faecal samples were examined for helminthes eggs by the simple floatation technique using saturated salt solution as the floating medium [4]. Examination of Giemsa stained blood smears and buffy coat smears was carried out as described by Nemi [5] to detect haemoparasites. Tests conducted to elucidate the presence of bacterial and parasitic causative agents did not reveal any significant finding.

## 3. Discussion

Peeling sweet cassava before use reduces the chances of cyanide toxicity as the peels of cassava varieties grown in Nigeria contain 5–10 times the concentration of hydrocyanic acid (HCN) in the pulp of fresh material [6]. Boiling is known to reduce the HCN concentration to a nontoxic level in man and livestock so that the cassava peel or pulp is fed in substantial amounts [7]. The cassava pulp used was boiled before feeding the pigs and therefore the clinical signs and lesions observed could not be attributed to cyanide toxicity. Cassava toxicity in man in Nigeria has been associated with goiter [8, 9], neuropathy [10], and fatty liver [11] because of the cyanide content, but none of these were observed in the pigs.

Scopoletin (6-methoxy-7-hydroxycoumarin) has been found in large quantities in cassava products including garri and cassava flour. The level of scopoletin is not affected by treatments such as sun drying, refrigeration, storage, and cooking [12]. Scopoletin is rapidly absorbed from the gut and excreted in the urine. Sublethal oral doses of scopoletin have been shown to increase the bleeding time in chicks [13] and to induce prolongation of blood clotting time [14]. These might explain the nonclotting of the blood observed in the pigs. Scopoletin is absorbed by erythrocytes in which it binds to the haemoglobin and affects erythrocyte structure and oxygen capture and delivery by haemoglobin [14]. The haemolysis of the red blood cells arising from this could be responsible for the icterus observed in the pigs.

Scopoletin is absorbed by mitochondria and it uncouples oxidative phosphorylation at low concentrations and directly inhibits it terminally at the cytochrome oxidase level [15]. This effect on the subcellular organelles of muscle cells could lead to paleness, degeneration, and necrosis of the fibres. This was observed histopathologically. Scopoletin is a neuromuscular blocking agent [16]. This action along with the changes in the muscle fibres could have been responsible for the clinically observed reluctance to move and the muscular weakness. Scopoletin initiates toxic liver damage at very low concentrations [17, 18]. This could account for the hepatic necrosis observed with resultant icterus. From the above observations one is tempted to suggest that the clinicopathology observed in the pigs fed peeled and boiled cassava pulp might be due to the effects of scopoletin, a noncyanide toxic principle in cassava. This should be investigated further. However, vitamin E/selenium responsive disease in pigs should also be considered as a differential diagnosis from the pathology but some of the clinical signs of vitamin E/selenium deficiency in pigs are different from those of the suspected scopoletin toxicity.
